# The applicability of established clinical and histopathological risk factors for tumor recurrence during long-term postoperative care in meningioma patients

**DOI:** 10.1007/s10143-021-01697-w

**Published:** 2021-11-20

**Authors:** Swenja Lüthge, Dorothee Cäcilia Spille, Andrea Ulrike Steinbicker, Stephanie Schipmann, Eileen Maria Susanne Streckert, Katharina Hess, Oliver Martin Grauer, Werner Paulus, Walter Stummer, Benjamin Brokinkel

**Affiliations:** 1 Department of Neurosurgery, University Hospital Münster, Albert-Schweitzer-Campus 1, Building A1, 48149 Münster, Germany; 2 Department of Anesthesiology, Intensive Care and Pain Medicine, University Hospital, Münster, Germany; 3 Institute of Neuropathology, University Hospital Münster, Münster, North Rhine Westphalia Germany; 4 Department of Pathology, University Hospital Schleswig-Holstein, Campus Kiel, Kiel, Germany; 5 Department of Neurology, University of Münster, Münster, Germany

**Keywords:** Meningioma, Long-term, Microsurgery, Progression, Recurrence, Resection, Simpson

## Abstract

Risk factors to predict late-onset tumor recurrence in meningioma patients are urgently needed to schedule control intervals during long-term follow-up. We therefore analyzed the value of established risk factors for postoperative meningioma recurrence for the prediction of long-term prognosis. Correlations of clinical and histopathological variables with tumor relapse after 3, 5, and 10 years following microsurgery were analyzed in uni- and multivariate analyses, and compared to findings in the entire cohort. In the entire cohort (*N* = 1218), skull base location (HR: 1.51, 95%CI 1.05–2.16; *p* = .026), Simpson ≥ IV resections (HR: 2.41, 95%CI 1.52–3.84; *p* < .001), high-grade histology (HR: 3.70, 95%CI 2.50–5.47; *p* < .001), and male gender (HR: 1.46, 95%CI 1.01–2.11; *p* = .042) were independent risk factors for recurrence. Skull base location (HR: 1.92, 95%CI 1.17–3.17; *p* = .010 and HR: 2.02, 95%CI 1.04–3.95; *p* = .038) and high-grade histology (HR: 1.87, 95%CI 1.04–3.38; *p* = .038 and HR: 2.29, 95%CI 1.07–4.01; *p* = .034) but not subtotal resection (HR: 1.53, 95%CI .68–3.45; *p* = .303 and HR: 1.75, 95%CI .52–5.96; *p* = .369) remained correlated with recurrence after a recurrence-free follow-up of ≥ 3 and ≥ 5 years, respectively. Postoperative tumor volume was related with recurrence in general (*p* < .001) but not beyond a follow-up of ≥ 3 years (*p* > .05). In 147 patients with a follow-up of ≥ 10 years, ten recurrences occurred and were not correlated with any of the analyzed variables. Skull base tumor location and high-grade histology but not the extent of resection should be considered when scheduling the long-term follow-up after meningioma surgery. Recurrences ≥ 10 years after surgery are rare, and predictors are lacking.

## Introduction

Despite sufficient local tumor control after microsurgery for intracranial meningiomas, recurrences are commonly observed and occur both early and within long-term follow-up. Remarkably, recommendations for radiological and clinical follow-up intervals during perioperative care of meningiomas have been focused on the early postoperative phase, while references for long-term follow-up are sparse [5, 6].

Currently, only a few clinical and histopathological/molecular variables are available to estimate the risk of postoperative tumor recurrence, and to enable a more personalized care of meningioma patients. Aside from histopathological grading [16], molecular characteristics such as hTERT promoter mutations [20], CDKN2A/B deletions [24], or DNA methylation pattern [21] have been shown to strongly correlate with prognosis and are increasingly utilized to further estimate the risk of postoperative tumor recurrence in daily clinical practice. Most notably, these characteristics usually reflect aggressive biological behavior and are therefore associated with *early* postoperative tumor recurrence, while molecular predictors for long-term prognosis remain sparse.

Among clinical variables, the Simpson grading system [25] has been widely established to assess the extent of resection intraoperatively, and, correspondingly, to further estimate the risk of postoperative tumor recurrence according to the amount of tumor tissue left behind after surgery [27, 29]. As the consequence, the Simpson grading system is frequently applied when indicating adjuvant irradiation therapy or scheduling follow-up intervals. However, recent studies increasingly discuss fundamental shortcomings of the Simpson classification system, including both the assessment by the neurosurgeon and its value for the prediction of prognosis [22]. Moreover, the value of the Simpson grading system for the prediction of *long-term* tumor control is largely unexplored, and most analyses are restricted to small cohorts and/or patients with tumors in distinct locations [12, 17, 19, 28].

In this study, we therefore provide integrative analyses for risk factors for late-onset postoperative tumor progression in a large-scale single-center series, and further compare the prognostic value of established clinical and histopathological variables during short- and long-term follow-up.

## Materials and methods

### Data collection

Clinical and histopathological data of all patients who underwent surgery for primary diagnosed intracranial meningioma between 1991 and 2021 in our department was obtained from the Münster Meningioma Database, as described previously [1, 3, 4, 8, 9, 27, 29]. Surgery had been indicated for any space-occupying or progressive and/or symptomatic lesions not feasible for radiosurgical treatment in the absence of contraindications against either surgery or anesthesia. Maximum safely achievable resection was performed in all patients. Adjuvant radiotherapy was recommended in all patients with histopathological diagnosed WHO grade III meningioma and in the case of subtotally resected atypical meningiomas as well as for grade I lesions after simple surgical decompression. Adjuvant chemotherapy was not administered. Postoperative volumetry had been performed for a previous study using a commercial neuronavigation software (Brainlab 2.6 Neuronavigation System, Brainlab AG, Munich, Germany), and considered the first available T1-weighted, contrast-enhanced images obtained within 6 months after surgery [27].

Clinical data was obtained from medical records and included patients’ age, sex, tumor location, the extent of resection according to the Simpson classification as determined by the attending neurosurgeon, the preoperative Karnofsky Performance Status Scale (KPS) [10], and the administration of adjuvant radiotherapy. Tumor location was classified into the following categories: falx cerebri/parasagittal, convexity, skull base, posterior fossa, and intraventricular. Histopathological grading and tumor subtype were diagnosed according to the current 2016 WHO criteria in all cases [16]. Hence, brain invasion was light-microscopically evaluated on hematoxylin and eosin and Elastica van Gieson–stained slides and diagnosed in the case of “irregular, tongue-like protrusions of tumor cells infiltrating underlying parenchyma, without an intervening layer of leptomeninges.”

Initial routine postoperative gadolinium-enhanced MRI was generally scheduled at 3 months after surgery and repeated annually and semi-annually in grade I and high-grade (WHO grade II/III) meningiomas, respectively. After 5 years of a progression-free follow-up, imaging was repeated every 2 years in grade I, yearly in grade II, and semi-annually in grade III lesions [5]. In patients with contraindications against MRI, contrast-enhanced CT-scans were performed for surveillance. Imaging was analyzed for progression by a team of two independent observers, including one neurosurgeon and one neuro-radiologist, and data about progression was additionally updated by standardized questionnaires, which were sent to the primary care takers. Progression-free survival (PFS) was determined as the duration between surgery and radiologically confirmed tumor recurrence or, in the case of an event-free follow-up, the date of last follow-up. Data collection and scientific use were approved by the local ethics committee (Ärztekammer Westfalen-Lippe, 2018–061-f-S) and approved by the patients in each single case.

### Statistical analyses

Data was described by standard statistics. Hence, categorical variables were characterized by absolute and relative frequencies and compared by Fisher’s exact test; continuous variables were described by median and range and compared by Mann–Whitney’s *U* test. Logistic regression modelling was used to calculate hazard ratios (HR) for categorical and continuous variables. For statistical analyses, tumor location and the extent of tumor resection were dichotomized in skull base vs non-skull base and GTR vs STR (Simpson ≥ IV) whenever indicated in the manuscript body. Time-to-progression analyses were performed using Kaplan–Meier curves and compared by log-rank tests. For uni- and multivariate analyses, Cox proportional hazard models and logistic regression were used and described with hazard ratio (HR), as well as with backward Wald’s *p*-values and 95% confidence intervals (CI). The following variables were included in multivariate analysis: age, sex (female (ref, reference) vs male), WHO grade (classified into grade I (ref) vs grade II /III), tumor location (classified as skull base vs non-skull base = ref), and the extent of resection (classified into STR vs GTR = ref). *P*-values < 0.05 were considered statistically significant throughout the whole analyses. All reported *p*-values are two-sided. IBM SPSS Statistics 27.0 (IBM, Armonk, NY, USA) was used for statistical analyses.

## Results

**Fig. 1:**
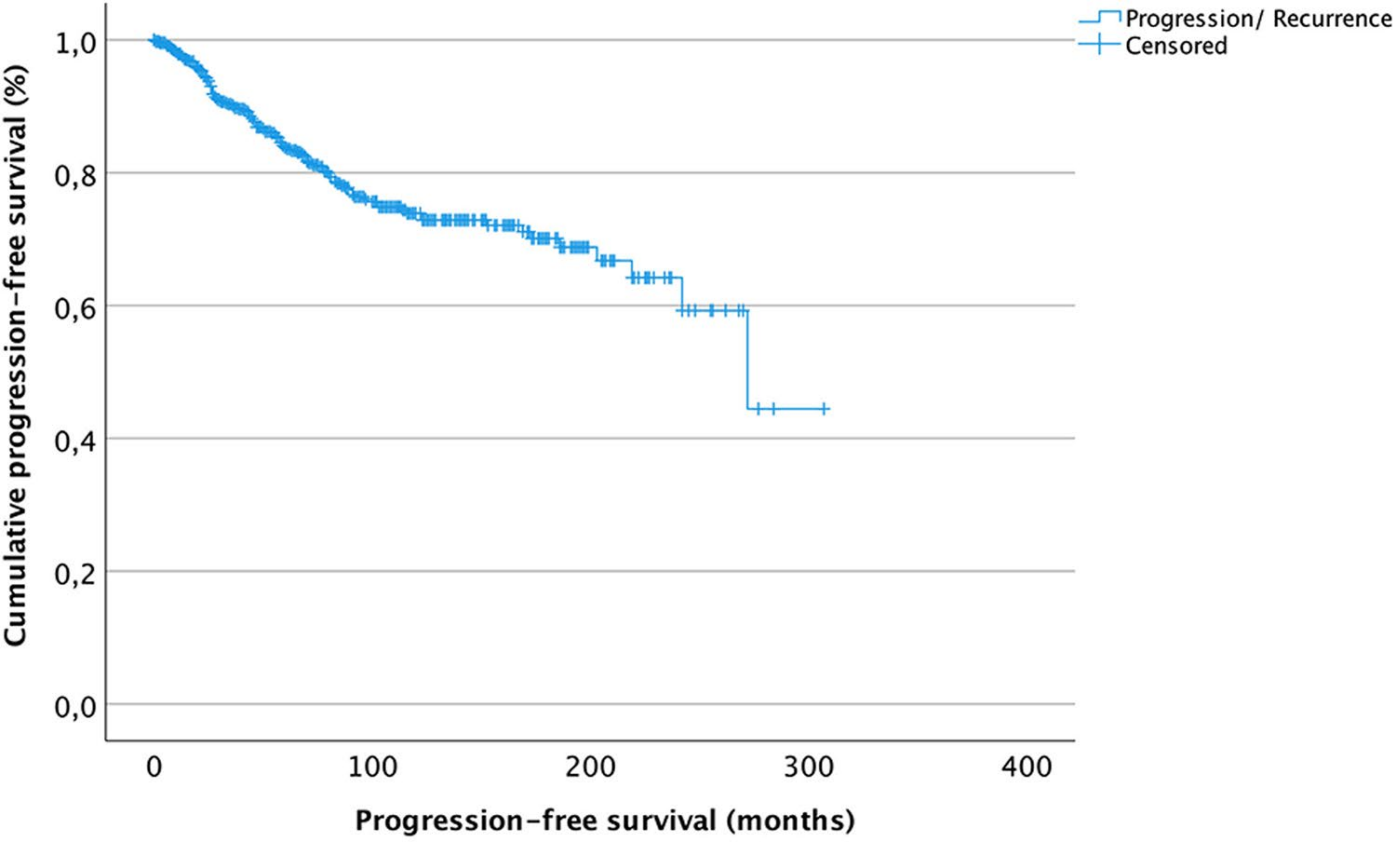
Kaplan–Meier plot displaying the progression-free survival of the entire cohort. Most recurrences were observed within short- and median-term follow-up, with progression-free survivals of 90%, 84%, 74%, and 70% after 3, 5, 10, and 15 years after microsurgery, respectively

Of 1517 surgeries performed for histopathologically confirmed meningiomas in our department between 1991 and January 2021, 97 (6%) spinal as well as 202 (13%) recurrent meningioma cases were excluded. The remaining 1218 cases were subjected to further statistical analyses. Table 1 summarizes baseline clinical and histopathological characteristics. Among 1075 grade I tumors, neuropathological analyses revealed meningothelial (*N* = 633, 59%), transitional (*N* = 321, 30%), fibrous (*N* = 50, 5%), angiomatous (*N* = 14, 1%), microcystic (*N* = 9, 1%), psammomatous (*N* = 1, < 1%), and secretory (*N* = 45, 4%) subtypes, while subtype was not further determinable in 2 cases (< 1%). Within a median follow-up of 29 months (range 0–307 months), recurrence was observed in 141 individuals (12%) after a median PFS of 36 months. Progression-free survival among the entire cohort at 3, 5, 10, and 15 years after microsurgery was 90%, 84%, 74%, and 70%, respectively (Fig. 1).

**Table 1:** Patients’ characteristics. Complete clinical and histopathological data was available in the vast majority of the analyzed 1218 patients

Variable	*N* (%)	Available data (*n*%)
Sex		1218 (100%)
Male	337 (28%)	
Female	881 (72%)	
Age (years; median, range)	59; 10–86	1218 (100%)
Preoperative KPS^a^ (mean, range)	80; 10–100	1198 (98%)
Extent of resection/Simpson grade		1146 (94%)
I	336 (28%)	
II	528 (43%)	
III	120 (10%)	
IV	158 13%)	
V	4 (< .5%)	
WHO grade		1218 (100%)
I	1075 (88%)	
II/III	143 (12%)	
Brain invasion	71 (6%)	1218 (100%)
Tumor location		1218 (100%)
Convexity	429 (35%)	
Falx/parasagittal	161 (13%)	
Skull base	542 (45%)	
Intraventricular	11 (1%)	
Posterior fossa	75 (6%)	
Adjuvant irradiation	58 (6%)	985 (81%)

^a^*KPS* Karnofsky Performance Score

Among the entire population, male gender (HR: 1.96, 95%CI 1.40–2.73; *p* < 0.001) and high-grade histology (HR: 3.23, 95%CI 2.26–4.60; *p* < 0.001) were correlated with an increased risk of recurrence. Risk of recurrence was also higher after Simpson grade II (HR: 1.81, 95%CI 1.12–2.91; *p* = 0.015), grade III (HR: 1.92, 95%CI 1.08–3.42; *p* = 0.027), or grade IV resections (HR: 3.45, 95%CI 1.95–6.12; *p* < 0.001) than after Simpson grade I surgery. Correspondingly, Simpson grade IV and V resections (subtotal resection, STR) were associated with a more than twofold risk of recurrence as compared to Simpson grade ≤ III surgeries (gross total resection, GTR, HR: 2.26, 95%CI 1.46–3.52; *p* < 0.001). Among the different tumor locations, only skull base lesions were associated with tumor relapse (HR: 1.78, 95%CI 1.19–2.65; *p* = 0.005). While no correlation was found between recurrence and brain invasion (OR: 1.49, 95%CI 0.88–2.52; *p* = 0.140), the presence of other grading criteria on the analyzed microscopic slides was strongly associated with tumor relapse (HR: 4.79, 95%CI 3.28–7.01; *p* < 0.001). Multivariate analyses confirmed skull base location (HR: 1.51, 95%CI 1.05–2.16; *p* = 0.026), Simpson ≥ IV resections (HR: 2.41, 95%CI 1.52–3.84; *p* < 0.001), high-grade histology (HR: 3.70, 95%CI 2.50–5.47; *p* < 0.001), and male gender (HR: 1.46, 95%CI 1.01–2.11; *p* = 0.042) as independent risk factors for tumor recurrence.

**Fig. 2:**
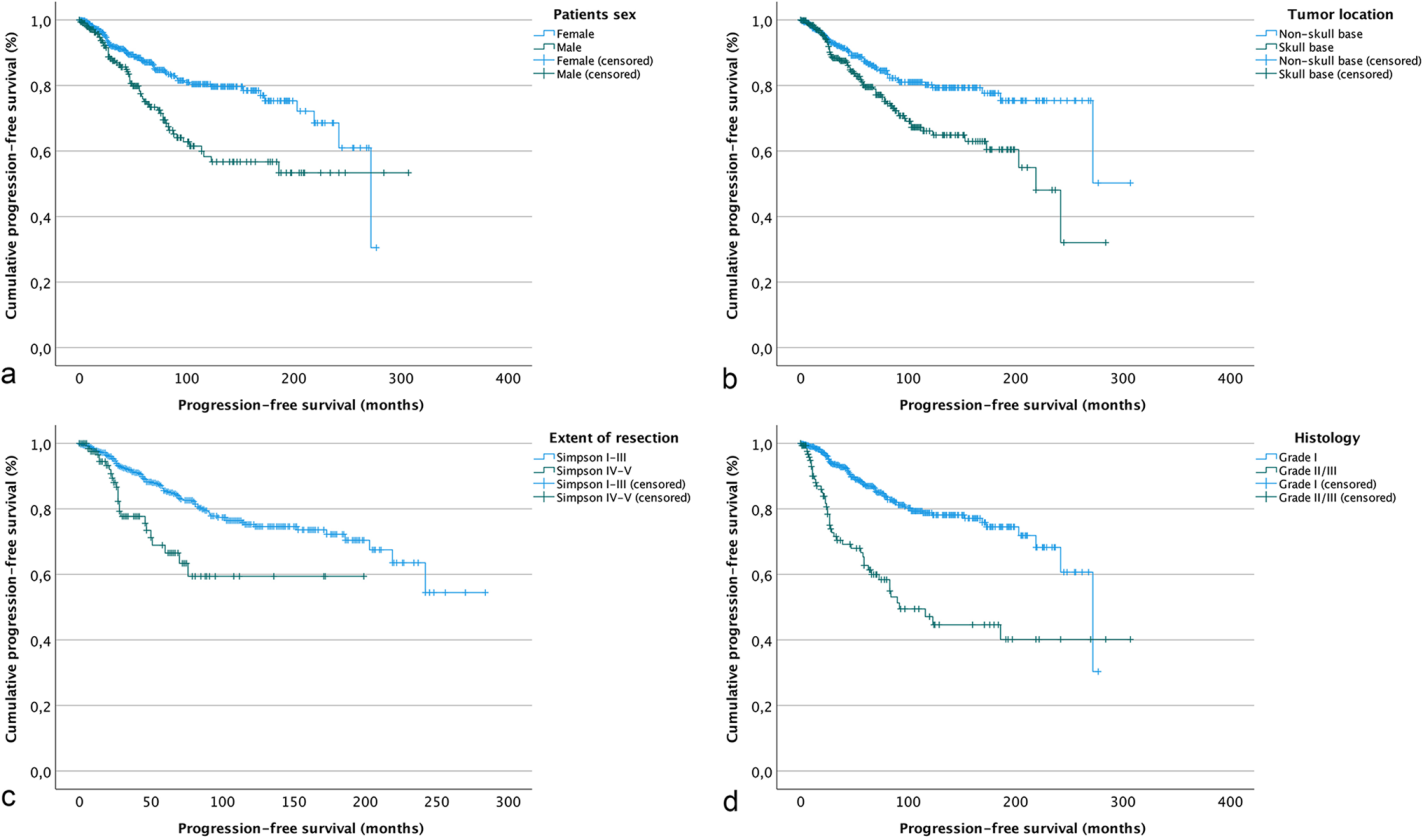
Kaplan–Meier plots of the progression-free survival in relation to clinical and histological variables. Overall PFS was significantly correlated with patients’ sex (**a**, *p* < .001), tumor location (**b**, *p* = .001), the extent of resection (**c**, *p* < .001), and histology (**d**, < .001, log-rank test, each). However, during median- and long-term follow-up, only sex, tumor location, and histology remained associated with prognosis

Subsequently, subgroup analyses were performed to further elucidate risk factors for tumor progression during median- and long-term follow-up. Here, despite sufficient cohort sizes, established clinical and histopathological risk factors for tumor progression as confirmed in analyses of the entire collective did not remain constantly correlated with recurrence (Fig. 2).

In detail, in 485 patients with a PFS of at least 3 years, recurrence was observed in 70 individuals (15%). In univariate analyses, male sex (HR: 2.31, 95%CI 1.45–3.69; *p* < 0.001), skull base tumor location (HR: 2.09, 95%CI 1.30–3.36; *p* = 0.002), and high-grade histology (HR: 1.88, 95%CI 1.08–3.26; *p* = 0.025) remained risk factors for progression development, while STR did not remain correlated with relapse. This also held true in multivariate analyses (Table 2) and when not dichotomizing the extent of resection (*p* > 0.05, data not shown).

**Table 2:** Risk factors for tumor recurrence during short- and long-term follow-up. Uni- and multivariate analyses confirmed well-established risk factors for tumor recurrence among the entire cohort. In contrast, the extent of resection did not remain a risk factor for progression beyond an event-free follow-up of 3 years. In 147 patients with an even-free course of at least 10 years, none of the analyzed variables remained a risk factor for tumor progression

	Variable	Univariable analysis: HR^a^ (95%CI), *p*-value	Multivariable analysis: HR (95%CI), *p*-value
Entire (*N* = 1218) cohort	Sex: male vs female (ref)	1.96 (1.40–2.73), *p* < .001	1.46 (1.01–2.11), *p* = .042
	Age at surgery (in years)	1.00 (.99–1.02), *p* = .590	1.00 (.99–1.02), *p* = .515
	Tumor location: skull base vs others (ref^b^)	1.71 (1.22–2.39), *p* = .002	1.51 (1.05–2.16), *p* = .026
	WHO grade II/III vs I (ref)	3.23 (2.26–4.60), *p* < .001	3.70 (2.50–5.47), *p* < .001
	Simpson grade ≥ IV vs I–III (ref)	2.26 (1.46–3.52), *p* < .001	2.41 (1.52–3.84), *p* < .001
≥ 36 months (*N* = 485) PFS^c^	Sex: male vs female (ref)	2.31 (1.45–3.69), *p* < .001	2.08 (1.24–3.48), *p* = .006
	Age at surgery (in years)	.99 (.97–1.01), *p* = .315	.99 (.97–1.01), *p* = .212
	Tumor location: skull base vs others (ref)	2.09 (1.30–3.36), *p* = .002	1.92 (1.17–3.17), *p* = .010
	WHO grade II/III vs I (ref)	1.88 (1.08–3.26), *p* = .025	1.87 (1.04–3.38), *p* = .038
	Simpson grade ≥ IV vs I–III (ref)	1.64 (.74–3.63), *p* = .220	1.53 (.68–3.45), *p* = .303
≥ 60 months (*N* = 346) PFS	Sex: male vs female (ref)	2.03 (1.10–3.75), *p* = .024	1.84 (.92–3.70), *p* = .086
	Age at surgery (in years)	1.00 (.98–1.03), *p* = .812	1.00 (.97–1.03), *p* = .982
	Tumor location: skull base vs others (ref)	2.26 (1.21–4.19), *p* = .010	2.02 (1.04–3.95), *p* = .038
	WHO grade II/III vs I (ref)	2.03 (1.01–4.09), *p* = .047	2.29 (1.07–4.01), *p* = .034
	Simpson grade ≥ IV vs I–III (ref)	1.68 (.51–5.56), *p* = .393	1.75 (.52–5.96), *p* = .369
≥ 120 months (*N* = 147) PFS	Sex: male vs female (ref)	.56 (.12–2.66), *p* = .463	.95 (.88–1.03), *p* = .185
	Age at surgery (in years)	.99 (.93–1.05), *p* = .688	.93 (.14–6.32), *p* = .938
	Tumor location: skull base vs others (ref)	3.08 (.86–11.02), *p* = .083	3.28 (.64–16.95), *p* = .156
	WHO grade II/III vs I (ref)	.88 (.18–4.35), *p* = .873	1.54 (.28–8.47), *p* = .617
	Simpson grade ≥ IV vs I–III (ref)	n/ap^d^	n/ap

^a^Hazard ratio

^b^Reference

^c^Progression-free survival

^d^Not applicable for statistical reasons

Patients with a PFS of at least 5 years (*N* = 346) developed tumor relapse in 42 cases (12%). Among those, only skull base tumor location (HR: 2.26, 95%CI 1.21–4.19; *p* = 0.010) and high-grade histology (HR: 2.03, 95%CI 1.01–4.09; *p* = 0.047) remained risk factors for progression in univariate analyses. Both risk factors were also confirmed in multivariate analyses (Table 2).

In 146 patients with a PFS of at least 10 years, only ten tumor recurrences were observed. Among those, neither uni- nor multivariate analyses revealed correlations between tumor recurrence and any of the analyzed variables (Table 2). Table 3 displays the characteristics of the patients who developed tumor relapse after more than 10 years following meningioma surgery. The last tumor recurrence was observed in a female patient 272 months following resection of a WHO grade I posterior fossa meningioma.

**Table 3:** Characteristics of patients who developed recurrence following more than 10 years after surgery. Recurrences were not correlated with any of the analyzed variables. In eight of ten patients with available information, recurrence was only diagnosed on routine follow-up imaging but not due to neurological deterioration

No	Age (yrs)	Sex	Location	Simpson grade	WHO grade	Adjuvant irradiation	PFS^a^ (months)
1	50	Female	Skull base	n/a^b^	I	No	123
2	46	Male	Parasagittal	III	II	No	123
3	67	Female	Skull base	II	I	No	153
4	64	Female	Convexity	n/a	I	No	169
5	38	Female	Skull base	III	I	No	173
6	46	Male	Convexity	I	II	No	186
7	50	Female	Skull base	I	I	No	203
8	45	Female	Skull base	III	I	Yes	219
9	42	female	Skull base	II	I	No	242
10	64	Female	Post. fossa	n/a	I	No	272

^a^Progression-free survival

^b^Not available

In subgroup analyses of 419 patients with available postoperative tumor volume as visualized on MRI within 6 months after surgery, median remnant volume was 0.00 ml (0.00–78.5 ml) and strongly correlated with recurrence (*p* < 0.001, previously published data [27]). However, no correlation between the postoperative tumor volume was found in 62 patients with available data (median tumor volume 0.00 ml, range 0.00–61.0 ml) and an event-free survival of at least 36 months after surgery (HR: 0.99, 95%CI 0.84–1.17; *p* = 0.898).

## Discussion

The postoperative recurrence rate in our series was 12% and was comparable to observations in previous studies [14, 15, 18]. As expected, both uni- and multivariate analyses in our series confirmed several well-established risk factors for postoperative meningioma recurrence in general. However, these correlations were not constantly confirmed during median-term and long-term follow-up.

Most notably, although found a strong risk factor for recurrence during short-term follow-up, the extent of resection was not related with tumor relapse during medium- and long-term prognosis. In fact, after an event-free follow-up of 3 years, no further correlation between the extent of resection and the risk of postoperative tumor relapse was found. This also held true in multivariate analyses, where other risk factors for recurrence remained largely stably correlated with tumor relapse, at least following the first 5 years after surgery. Remarkably, similar findings were found when analyzing the prognostic value of the postoperative tumor volume. Despite the limited number of patients in this subcohort, these findings clearly support the thesis of a minor prognostic role of the extent of resection for long-term tumor control in meningioma patients. Within the last years, several studies revealed shortcomings of the Simpson classification system and raised doubts about its prognostic value for the estimation of the risk of postoperative tumor recurrence [22]. Recent series reported a less favorable extent of resection in a considerable portion of meningioma patients comparing the intraoperatively assessed Simpson grade with results from postoperative MRI [26, 27]. Furthermore, the prognostic value of the extent of resection appears to be related to the tumor location [29]. In an own series, we demonstrated a higher prognostic value of the postoperative tumor volume than of the extent of resection according to the Simpson classification system [27]. In contrast, reports about the long-term prognostic value of the extent of resection are sparse and restricted to small series of tumors at distinct locations [12, 17, 19]. *Pettersson-Segerling *et al*.* reported higher risks of tumor relapse after subtotal (Simpson grade IV/V) than after gross total resection 10 and 25 years after surgery in a series of 51 patients with parasagittal meningiomas, but without giving *p*-values to reveal statistical significance or providing integrative analyses of other risk factors [17]. In contrast to our series, *Gousias* and colleagues reported the Simpson grade to be both a predictor for overall and long-term (> 5 years) risk of recurrence in a series of 901 meningioma patients [7]. Hence, although the Simpson grading system remains a valuable tool to quantify the extent of resection intraoperatively, our findings further challenge its prognostic value.

Higher recurrence rates in skull base lesions have been reported previously and might reflect distinct genetic alterations [31], such as AKT1E17K mutations [30], or higher rates of subtotal resections, and, similarly, more residual tumor remnants left behind after surgery [27]. In fact, skull base tumor location in our series was associated with both subtotal resection as well as higher postoperative tumor volumes as compared to non-skull base lesions (*p* < 0.001, each, data not shown). As described above, previous analyses revealed a strong correlation of the postoperative tumor volume with tumor relapse after meningioma surgery [27].

Similarly, previous analyses reported higher recurrence rates in male than in female meningioma patients [32], e.g., due to higher rates of high-grade lesions among the first [23]. Although, correspondingly, rates of grade II/III histology were more than twofold higher in male than in female patients (21% vs 8%, *p* < 0.001) in our series, correlation with recurrence was also confirmed in WHO grade–adjusted multivariate analyses. Hence, further characteristics, such as endocrine mechanisms or sex-associated genetic alterations [31], might additionally contribute to the worse prognosis of male patients in our series.

High-grade histology according to the 2016 classification of brain tumors remained a strong and independent risk factor for progression within the first 10 years following microsurgery. Over the last years, studies on molecular alterations in meningiomas identified a number of strong risk factors for postoperative tumor relapse [2]. However, with few exceptions, these variables mostly predicted recurrences within the first 3 to 5 years after surgery [13, 20, 24], while molecular predictors for medium- and long-term prognosis are sparse [11, 21]. Hence, despite the immense value of molecular alterations for the prediction of short-term prognosis and, eventually, for indicating adjuvant treatment, applicability to further individualize medium- and long-term follow-up is limited. On the other hand, our findings clearly underline the value of the established histopathological analyses and diagnosis in face of an increasing implication of molecular alterations during meningioma diagnostics and research.

Beyond an event-free follow-up of at least 10 years, only < 1% of the included patients suffered tumor relapse, and none of the previously identified risk factors for prognosis remained correlated with recurrence. While eventually explainable by the low number of tumor relapses (*N* = 10) in this subcohort (*N* = 147), the lack of any correlation even in univariate analyses is remarkable. Correspondingly, during visual inspection of the characteristics of patients suffering from late-onset recurrence, no variable is obvious (Table 3). All recurrences were identified based on regular follow-up imaging but not due to new or progressive symptoms. While therefore high-grade histology and skull base tumor location should both be considered when attempting further characterization of follow-up intervals and duration beyond an event-free interval of 5 years, determination of the risk of long-term tumor relapse is hardly possible.

Although providing integrative analyses in a large series, the authors are aware of some limitations of the study. In fact, our study suffers the limitations of its retrospective nature, and the single-center character further limits general transferability. Due to the long inclusion period, details about adjuvant irradiation, especially indications, modality, and timing, were hardly available and could not be considered satisfactory for statistical analyses. However, considering the low number of patients receiving adjuvant irradiation (6%), the potential bias is supposed to be minor. Moreover, due to the large volume of the series, molecular characteristics, e.g., hTERT promoter mutations or DNA methylation pattern, have not been analyzed.

In conclusion, skull base tumor location and high-grade histology were confirmed as strong and independent risk factors for tumor relapse following ≥ 5 years after surgery and should be considered when revising the duration of follow-up and imaging intervals in meningioma patients. In contrast, the prognostic value of the extent of resection beyond an event-free course of 3 years is doubtful. As our findings were consistent in both uni- and multivariate analyses and were additionally confirmed by volumetry in a subcohort, we recommend not to consider the extent of resection when scheduling follow-up intervals in meningioma patients after an event-free follow-up of ≥ 3 years. Tumor relapses following more than 10 years after surgery are very rare, and corresponding predictors are lacking.

## Data Availability

Not provided.
